# Supplementary material for: Fermentation of Betaphycus gelatinum Using Lactobacillus brevis: Growth of Probiotics, Total Polyphenol Content, Polyphenol Profile, and Antioxidant Capacity
Source: Foods. 2023 Sep 6;12(18):3334. doi: 10.3390/foods12183334 (PMC10527574; doi:10.3390/foods12183334)
Supplement: Supplementary file 1 [file foods-12-03334-s001.zip › foods-2545913-supplementary.pdf]

Supplementary Materials

Supporting Information

Fermentation of Betaphycus gelatinum using Lactobacillus brevis: Growth of probiotics, total polyphenol content, polyphenol profile, and antioxidant capacity

Zhe Wang [1], Caibo Zhao [1], Zhiqiang Guo [2], Shuyi Li [3], Zhenzhou Zhu [3], Nabil Grimi [4], Juan Xiao [1]

[1] Hainan Engineering Research Center of Aquatic Resources Efficient Utilization in South China Sea, Key 6
Laboratory of Seafood Processing of Haikou, Key Laboratory of Food Nutrition and Functional Food of 7
Hainan Province, School of Food Science and Engineering, Hainan University, Haikou 570228, China 8

[2] School of Marine Science and Engineering, Hainan University, Haikou 570228, China 9

[3] National R&D Center for Se-rich Agricultural Products Processing, Hubei Engineering Research Center for 10
Deep Processing of Green Se-rich Agricultural Products, School of Modern Industry for Selenium Science 11
and Engineering, Wuhan Polytechnic University, Wuhan 430023, PR China 12

[4] Sorbonne University, Université de Technologie de Compiègne, ESCOM, EA 4297 TIMR, Centre de recher- 13
che Royallieu - CS 60319, 60203 Compiègne Cedex, France 14

* Correspondence: xiaojuan209218@163.com; Tel.: +86-898-66193581 15

Contents of Supporting Information

Table S1. Calibration curves of different standards. The abscissa of the calibration curves is the concentration of the standard (ng/mL) and the ordinate is the peak area.

No.	Tentative assignment	Calibration curves
1	4-hydroxybenzoic acid	Y=1503.70X+174275 (R^2=0.997)
2	ethyl vanillin	Y=33.74X-3891.10 (R^2=0.997)
3	trans-cinnamic acid	Y=17.90X+1955.30 (R^2=0.998)
4	caffeic acid	Y=1761X+285632 (R^2=0.996)
5	ferulic acid	Y=2.09X+113.15 (R^2=0.995)
6	rosmarinic acid	Y=21.27X+9103.20 (R^2=0.998)
7	1,3-O-dicaffeoylquinic acid	Y=509.86X+20726 (R^2=0.997)
8	gallocatechin	Y=29.82X+1255.70(R^2=0.997)
9	epigallocatechin	Y=37.93X-1499.20(R^2=0.999)
10	epicatechin gallate	Y=109.66X-3000.70 (R^2=0.996)
11	ellagic acid	Y=71.27X-8677.40 (R^2=0.997)
12	quercetin	Y=430.42X+53041 (R^2=0.996)
13	morin	Y=118.15X+22474 (R^2=0.994)
14	myricetin	Y=30.68X+21764(R^2=0.997)
15	taxifolin	Y=441.29X+49173 (R^2=0.999)
16	hinokiflavone	Y=23.25X+11105 (R^2=0.997)
17	hyperoside	Y=121.78X-10959 (R^2=0.996)
18	isoquercitrin	Y=107.58X-8109.10 (R^2=0.998)
19	kaempferol-3-O-glucoside	Y=345.55X-36418 (R^2=0.999)
20	cosemetin	Y=304.80X+14250 (R^2=0.995)
21	vitexin	Y=2.11X-25.15 (R^2=0.996)
22	myricitrin	Y=151.48X-15341 (R^2=0.999)
23	taxifolin-7-O-rhamnoside	Y=529.35X+71370 (R^2=0.997)
24	isorhamnetin-3-O-glucoside	Y=102.40X-17827 (R^2=0.995)
25	cyanidin	Y=4.47X+1099.20 (R^2=0.9997)
26	2,4-dibromophenol	Y=42.42X-400 (R^2=0.996)
27	2,4,6-tribromophenol	Y=2.80X+36.48 (R^2=0.990)

Note: All standards were determined at gradients of 313, 625, 1250, 2500, and 5000 ng/mL.

Table S2. Pearson correlation coefficient among the free, bound and the sum of free and bound TPC and antioxidant activity of *Betaphycus gelatinum* substrate fermented by *L. brevis* for 0 h and 60 h.

	TPC-F	TPC-B	TPC-S	FRAP-F	FRAP-B	DPPH-F	DPPH-B	ABTS-F	ABTS-B
TPC-F	**1**								
TPC-B	**-0.84914**	**1**							
TPC-S	0.57605	-0.05742	**1**						
FRAP-F	**0.99359**	-0.78406	0.66466	**1**					
FRAP-B	-0.62114	**0.81563**	0.08821	-0.55843	**1**				
DPPH-F	**0.98081**	-0.74091	0.70728	**0.99463**	-0.56363	**1**			
DPPH-B	**-0.88736**	**0.98302**	-0.15593	**-0.83339**	**0.87498**	-0.80821	**1**		
ABTS-F	**0.9679**	-0.68927	0.76281	**0.99**	-0.4582	**0.9921**	-0.74812	**1**	
ABTS-B	**-0.93136**	**0.97807**	-0.24678	**-0.8858**	**0.81873**	**-0.8591**	**0.99331**	**-0.81158**	**1**

Note: TPC: total phenolic content, TPC-F: free TPC; TPC-B: bound TPC; TPC-S: the sum of free and bound TPC; FRAP-F: FRAP of free polyphenol extracts; FRAP-B: FRAP of bound polyphenol extracts; DPPH-F: DPPH radical scavenging capacities of free polyphenol extracts; DPPH-B: DPPH radical scavenging capacities of bound polyphenol extracts; ABTS-F: ABTS radical scavenging capacities of free polyphenol extracts; ABTS-B: ABTS radical scavenging capacities of bound polyphenol extracts.
